# Large-scale M1 microcircuit model with plastic input connections from biological PMd neurons used for prosthetic arm control

**DOI:** 10.1186/1471-2202-16-S1-P153

**Published:** 2015-12-18

**Authors:** Salvador Dura-Bernal, Cliff C Kerr, Samuel A Neymotin, Bejamin A Suter, Gordon MG Shepherd, Joseph T Francis, William W Lytton

**Affiliations:** 1 Department of Physiology and Pharmacology, SUNY Downstate, Brooklyn, NY 11203, USA; 2 School of Physics, University of Sydney, Sydney, NSW, Australia; 3 Department Physiology, Northwestern University, Chicago, IL, 60611, USA

## 

**Figure 1:**
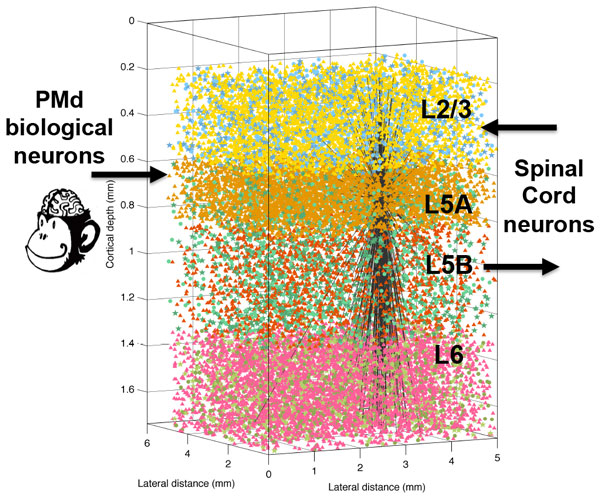
**Model of primary motor cortex showing inputs from PMd biological neurons, and closed-loop interaction with virtual musculoskeletal arm**. Cell types are shown with different colors. The output connections of a single L5A neuron are shown to illustrate connectivity.

We have developed a hybrid model of motor control for a brain-machine interface that is based on a large-scale model of primary motor cortex (M1) based on several mammalian studies (Figure 1). The M1 model consisted of 10,000 spiking Izhikevich neurons of four types: regular-firing and bursting pyramidal neurons, and fast-spiking and low-threshold-spiking interneurons. Within the M1 population, cell proportions, locations, connectivity and delays were drawn primarily from mouse experimental data. Properties were based on cell body cortical depth (distance from pia to white matter). Synapses included four different receptors: AMPA_, _NMDA_, _GABA_A _and GABA_B. _The model exhibited realistic physiological properties, including firing rates and local field potential spectra.

Pyramidal tract-projecting neurons in layer 5B were connected to a descending spinal cord neural population, which provided excitation to the muscles of a realistic virtual musculoskeletal arm. Proprioceptive feedback from the arm was encoded in an ascending spinal cord population which then projected to M1 layer 2/3. The virtual arm movements were also followed by a robotic arm [1].

An additional population, which reproduced the spiking patterns recorded from 92 neurons of macaque dorsal premotor cortex (PMd) during a center-out reaching task, was connected to M1 layer 5A providing a modulatory input [2].

The network was trained to drive the virtual arm to reach multiple targets, by combining arm exploratory movements with reinforcement learning and spike-timing dependent plasticity (STDP). Synaptic plasticity occurred between multiple populations, including between the PMd inputs and layer 5A neurons. Tuning of learning metaparameters was achieved by employing parallel evolutionary algorithms in a high-performance computing cluster.

This work moves towards a new generation of neuroprosthetic systems where biological brain circuits interact directly with biomimetic cortical models, and employ co-adaptation and learning to achieve a functional task. Such a multiscale approach, ranging from the cellular to the behavioral level, will provide deeper insights into brain dynamics and have applications for the diagnosis and restoration of brain disorders.
